# Supplementary material for: Mapping fast DNA polymerase exchange during replication
Source: Nat Commun. 2024 Jun 22;15:5328. doi: 10.1038/s41467-024-49612-3 (PMC11193749; doi:10.1038/s41467-024-49612-3)
Supplement: Supplementary file 1 — Supplementary Information [file 41467_2024_49612_MOESM1_ESM.pdf]

Supplementary Information for the article

Mapping fast DNA polymerase exchange during replication

Longfu Xu[1], Matthew T.J. Halma[1], Gijs J. L. Wuite[1*]

[1] Department of Physics and Astronomy and LaserLab, Vrije Universiteit Amsterdam, De Boelelaan 1081, 1081 HV, Amsterdam, The Netherlands

[*]To whom correspondence may be addressed: g.j.l.wuite@vu.nl

Supplementary Methods

The pKYB1 DNA construct

A simplified ssDNA/dsDNA construct designed to mimic the replication fork, comprising a single template and primer, was utilized to investigate the dynamics of DNA polymerase under controlled conditions (see **Supplementary Figure 4**). This construct was both end-labelled with biotin, following a modified version of established protocols[1]. Initially, 2-3 μg of the pKYB1 vector was digested in a 50 μL mixture containing high-fidelity restriction enzymes EcoRI-HF, KpnI-HF (NEB, 20 U/μL, in CutSmart Buffer), Biotin-14-dATP (Eurofins or Biolegio), dTTP (Thermo Scientific, 10297018), and Klenow enzyme (Thermo Scientific, EP0421), supplemented with digestion buffer and nuclease-free water. The mixture was incubated at 37°C for 1 hour. Post-digestion, purification was conducted using the Qiaquick protocol, which entailed PB buffer addition, centrifugation, PE buffer washes, and EB buffer elution. The digested product underwent ligation with a KpnI-Biotin oligo (25 μM, diluted in EB buffer) using ligation buffer and T4 ligase (Thermo Scientific, EL0014), incubating at room temperature for 2 hours. The ligation product was further purified with an Amicon filter, employing TE buffer (NaN_3 included) washes. A Nanodrop measured the concentration of the final construct, with a noted 40% DNA loss from washing.

This DNA construct was chosen for its ability to provide a controlled environment to study the fundamental processes of DNA polymerase dynamics, including rapid exchange, processivity, and interaction with ssDNA/dsDNA junctions.

Protein Construction, Expression, Purification and Labelling

The construction of a SNAP-tagged T7 DNA polymerase involved the integration of a SNAP tag at the N-terminus for fluorescent dye labeling and a 6xHis tag upstream for purification. T7 gp5 was cloned into pET-Duet1 vector's MCS1 (NcoI-NotI) and maintained in *E. coli* strain NEB10 Beta. The fused protein was expressed in Rosetta (DE3) strain in LB medium and verified using the Expasy Translation Tool and SDS-PAGE (**Supplementary Figure 5**). Subsequent experiments added a 2-4-fold molar excess of trxA to T7 DNA polymerase, following earlier protocols[2,3]. Our fluorescently labeled recombinant DNA polymerase showed activity comparable to commercial counterparts (**Supplementary Figures 6** and **7**).

The genetic transformation and protein expression protocol was conducted over four days. Initially, 1μl of pETDuet:N6xHisgp5 plasmid (~200ng/μl) was transformed into 50μl of Rosetta cells. The next day, colonies were selected and inoculated into 100ml LB medium with chloramphenicol and carbenicillin. On day three, OD600 measurements of 1/10 diluted starter cultures were taken before inoculating 4x400ml LB cultures per strain, adjusting OD600 to 0.080. After approximately 3 hours, when OD600 reached 0.5-0.6, cultures were conditioned to room temperature, followed by incubator cooling with ice to expedite temperature reduction. Subsequently, an additional round of antibiotics and IPTG (0.4mM final concentration) were added, with cultures left to grow overnight at room temperature. On the final day, cells were harvested by centrifugation at 9000xg for 20 minutes, washed with LB, and the cell pellets collected, weighed, and flash frozen in liquid nitrogen for storage at -20°C or -80°C.

Protein purification was conducted the following day to minimize degradation. All procedures were performed at 4°C or on ice, utilizing cold buffers with freshly added DTT, β-Mercaptoethanol, and protease inhibitors to ensure stability. The cell pellet was suspended in ice-cold lysis buffer at a ratio of 5ml per gram of pellet, ensuring a homogeneous suspension through gentle stirring with a clean glass spatula. Cell disruption was achieved using a Stansted cell disruptor. Centrifuge tubes were pre-chilled on ice for 20 minutes before use to maintain low temperatures during centrifugation at 29,000rpm (101,727xg) for 40 minutes using a Beckman JA 30.50Ti type centrifuge. The crude extract volume was measured post-centrifugation, informing the subsequent PEI precipitation step where PEI was added to a final concentration of 0.5%, stirred continuously at 4°C, and allowed to stand for 40 minutes before a second centrifugation under identical conditions. The supernatant was then treated with ammonium sulfate to a 65% saturation to precipitate T7 DNA polymerase, followed by centrifugation at 13,400rpm (27,000xg) for 30 minutes. The pellet was rinsed in 65% ammonium sulfate buffer, centrifuged again, and the resultant pellet dissolved in Talon binding buffer. This solution was dialyzed overnight against 2L of buffer, with at least one buffer change, then processed through a prepared Talon column. Post-incubation, the column was washed, and the protein eluted in six fractions, which were analyzed for protein concentration using a Nanodrop and Bradford Assay.

Fractions containing protein were concentrated using Amicon4 centrifuge filters. Subsequently, a 10-fold molar excess of SNAP-Surface® 549 dye was added to the protein sample and the conjugation reaction proceeded for three hours in the dark at room temperature. The mixture was then desalted with storage buffer containing 50% glycerol and dialyzed again overnight. The final protein concentration was determined using a Bradford assay, with the solution's purity verified by Nanodrop analysis. Finally, the sample was dialyzed into a storage buffer (50 mM Tris-HCl (pH = 7.4), 0.1 mM EDTA, 1 mM DTT and 50% glycerol) and stored at -20°C. The resulting degree of labelling was determined by absorbance to be ~60%. The labeled proteins were aliquoted, flash-frozen, and stored at -80°C.

Protein Structure Prediction and Activity Test

The protein structure of SNAP-DNA polymerase was predicted using the RoseTTAFold Modeling Method[4], obtained with a confidence score of 0.78 (see **Supplementary Figure 6**).

To validate the functionality of the SNAP-DNA polymerase, control experiments were conducted using two independent assays: real-time DNA primer extension assay and single-molecule assay. Comparative analyses were performed between commercial T7 DNA polymerase (NEB, # M0274L) without a SNAP-tag and our SNAP-tagged DNA polymerase. DNA polymerase experiments were performed in a standard measurement buffer containing 20 mM Tris-HCl pH 7.5, 100 mM NaCl, 3 mM $MgCl_2$, 1 mM DTT, and 0.02% BSA. DNA polymerase concentration was set at 30 nM unless specified otherwise. Note that the concentration was determined using a Bradford assay. We included a 2-4-fold molar excess of thioredoxin relative to the SNAP-DNA polymerase in our experiments[2,3].

Real-time DNA primer extension assay. The detailed method is based on previous publication with minor modifications[5]. Activity of T7 DNA polymerase was measured using a 42-nt long DNA template

annealed to a 30-nt primer strand. The carboxyfluorescein (6-FAM) moiety was located at the 5′ end of the template strand. Template strand: 5′/6-FAM/-CCC CCC CCC ATG CGC ACC TAA AGT TGG GAG TCC TTC GTC CTA-3′. Primer strand: 5′-TAG GAC GAA GGA CTC CCA ACT TTA GGT GCG-3′. Reactions were performed in a final volume of 10 µl with 25uM of each dNTP in buffer 20 mM Tris–HCl pH 7.5, 50 mM NaCl, 3mM $MgCl_2$, 1 mM DTT, 0.05% BSA and 0.05% Tween-20. Reactions were initiated by the addition of 70 nM labelled DNA to DNA polymerase with indicated concentration and measured in a 348-well plate using a BMG Labtech Pherastar FS plate reader during 20 min with 7 s intervals at 25°C. Data were analysed in GraphPad Prism and the rate was estimated by using the initial linear section of the curve (approximately the first 3 min) as shown in **Supplementary Figure 7A** and **7B**.

Single-molecule assays. We performed single-molecule experiments using a LUMICKS C-Trap instrument, integrating dual-trap optical tweezers, and a 5-channel microfluidic flow cell. Biotinylated pKYB1 DNA constructs were tethered in situ between two 1.76 µm streptavidin-coated microspheres (Spherotech Inc) within the flow cell. The presence of a single DNA molecule was confirmed via a change in the F-x curve. All experiments were conducted at room temperature, with the flow turned off during data acquisition. We analyzed the basepair versus time traces (from F-x curve) to identify changing points, which mark shifts in the polymerase (pol) or exonuclease (exo) activity. Initially, we mitigated the noise in the traces using a Savitzky-Golay (SG) filter with a window size of 15. The first derivative of these filtered traces was then calculated, serving as the basis for identifying significant changes in the pol or exo trend. To detect these changes, we applied a step-detection method used in prior research[6]. This allowed us to mark the steps or change points in the basepair-time traces. The processivity, velocity and duration are calculated for each segment. The results, illustrated in in **Supplementary Figure 7C** for the commercial enzyme[7] and **Supplementary Figure 7D** for the SNAP-tagged polymerase, indicate that the SNAP tag does not significantly impact the enzyme's activity or its DNA interaction, as both enzymes exhibited comparable kinetics.

Plasmid sequence of pETDuet-1_SNAP-DNA polymerase

The construction of SNAP-tagged T7 DNA polymerase is genetically engineered by attaching the SNAP tag at its N-terminus, allowing it to be labeled with various fluorescent dyes. To facilitate easy purification, a 6xHis tag was added to the SNAP tag's N-terminus. The expression of fused protein was verified using the Expasy Translation Tool and SDS-PAGE (**Supplementary Figure 5**).

Plasmid sequence and key sequence highlights include:

- Interested gene sequence in capitalized letter,
- 6XHis-SNAP tag in purple,
- flexible linker in red,
- gp5 gene in cyans,

ggggaattgtgagcggataacaattcccctctagaaataattttgtttaactttaagaaggagatataccatgggcagca

gccatcaccatcatcaccacagccaggatccgatg
GACAAAGATTGCGAAATGAAACGTACCACCCTGGATAGCCCGCTGGGCAAACTGGAACTGAGCG

GCTGCGAACAGGGCCTGCATGAAATTAAACTGCTGGGTAAAGGCACCAGCGCGGCCGATGCGG
TTGAAGTTCCGGCCCCGGCCGCCGTGCTGGGTGGTCCGGAACCGCTGATGCAGGCGACCGCG
TGGCTGAACGCGTATTTTCATCAGCCGGAAGCGATTGAAGAATTTCCGGTTCCGGCGCTGCATC
ATCCGGTGTTTCAGCAGGAGAGCTTTACCCGTCAGGTGCTGTGGAAACTGCTGAAAGTGGTTAA
ATTTGGCGAAGTGATTAGCTATCAGCAGCTGGCGGCCCTGGCGGGTAATCCGGCGGCCACCGC
CGCCGTTAAAACCGCGCTGAGCGGTAACCCGGTGCCGATTCTGATTCCGTGCCATCGTGTGGTT
AGCTCTAGCGGTGCGGTTGGCGGTTATGAAGGTGGTCTGGCGGTGAAAGAGTGGCTGCTGGCC
CATGAAGGTCATCGTCTGGGTAAACCGGGTCTG

ggc ggt ggt tct ggt ggt ggt ggt tct ggt ggt ggc ggt tcc atg

ATCGTTTCTGACATCGAAGCTAACGCCCTCTTAGAGAGCGTCACTAAGTTCCACTGCGGGGTTAT
CTACGACTACTCCACCGCTGAGTACGTAAGCTACCGTCCGAGTGACTTCGGTGCGTATCTGGAT
GCGCTGGAAGCCGAGGTTGCACGAGGCGGTCTTATTGTGTTCCACAACGGTCACAAGTATGACG
TTCCTGCATTGACCAAACTGGCAAAGTTGCAATTGAACCGAGAGTTCCACCTTCCTCGTGAGAAC
TGTATTGACACCCTTGTGTTGTCACGTTTGATTCATTCCAACCTCAAGGACACCGATATGGGTCTT
CTGCGTTCCGGCAAGTTGCCCGGAAAACGCTTTGGGTCTCACGCTTTGGAGGCGTGGGGTTATC
GCTTAGGCGAGATGAAGGGTGAATACAAAGACGACTTTAAGCGTATGCTTGAAGAGCAGGGTGA
AGAATACGTTGACGGAATGGAGTGGTGGAACTTCAACGAAGAGATGATGGACTATAACGTTCAG
GACGTTGTGGTAACTAAAGCTCTCCTCGAGAAGCTACTCTCTGACAAACATTACTTCCCTCCTGA
GATTGACTTTACGGACGTAGGATACACTACGTTCTGGTCAGAATCCCTTGAGGCCGTTGACATTG
AACATCGTGCTGCATGGCTGCTCGCTAAACAAGAGCGCAACGGGTTCCCGTTTGACACAAAAGC
AATCGAAGAGTTGTACGTAGAGTTAGCTGCTCGCCGCTCTGAGTTGCTCCGTAAATTGACCGAAA
CGTTCGGCTCGTGGTATCAGCCTAAAGGTGGCACTGAGATGTTCTGCCATCCGCGAACAGGTAA
GCCACTACCTAAATACCCTCGCATTAAGACACCTAAAGTTGGTGGTATCTTTAAGAAGCCTAAGA
ACAAGGCACAGCGAGAAGGCCGTGAGCCTTGCGAACTTGATACCCGCGAGTACGTTGCTGGTG
CTCCTTACACCCCAGTTGAACATGTTGTGTTTAACCCTTCGTCTCGTGACCACATTCAGAAGAAA
CTCCAAGAGGCTGGGTGGGTCCCGACCAAGTACACCGATAAGGGTGCTCCTGTGGTGGACGAT
GAGGTACTCGAAGGAGTACGTGTAGATGACCCTGAGAAGCAAGCCGCTATCGACCTCATTAAAG
AGTACTTGATGATTCAGAAGCGAATCGGACAGTCTGCTGAGGGAGACAAAGCATGGCTTCGTTA
TGTTGCTGAGGATGGTAAGATTCATGGTTCTGTTAACCCTAATGGAGCAGTTACGGGTCGTGCGA
CCCATGCGTTCCCAAACCTTGCGCAAATTCCGGGTGTACGTTCTCCTTATGGAGAGCAGTGTCG
CGCTGCTTTTGGCGCTGAGCACCATTTGGATGGGATAACTGGTAAGCCTTGGGTTCAGGCTGGC
ATCGACGCATCCGGTCTTGAGCTACGCTGCTTGGCTCACTTCATGGCTCGCTTTGATAACGGCG
AGTACGCTCACGAGATTCTTAACGGCGACATCCACACTAAGAACCAGATAGCTGCTGAACTACCT
ACCCGAGATAACGCTAAGACGTTCATCTATGGGTTCCTCTATGGTGCTGGTGATGAGAAGATTGG
ACAGATTGTTGGTGCTGGTAAAGAGCGCGGTAAGGAACTCAAGAAGAAATTCCTTGAGAACACC
CCCGCGATTGCAGCACTCCGCGAGTCTATCCAACAGACACTTGTCGAGTCCTCTCAATGGGTAG
CTGGTGAGCAACAAGTCAAGTGGAAACGCCGCTGGATTAAAGGTCTGGATGGTCGTAAGGTACA
CGTTCGTAGTCCTCACGCTGCCTTGAATACCCTACTGCAATCTGCTGGTGCTCTCATCTGCAAAC
TGTGGATTATCAAGACCGAAGAGATGCTCGTAGAGAAAGGCTTGAAGCATGGCTGGGATGGGGA
CTTTGCGTACATGGCATGGGTACATGATGAAATCCAAGTAGGCTGCCGTACCGAAGAGATTGCT
CAGGTGGTCATTGAGACCGCACAAGAAGCGATGCGCTGGGTTGGAGACCACTGGAACTTCCGG
TGTCTTCTGGATACCGAAGGTAAGATGGGTCCTAATTGGGCGATTTGCCACTGA GCGGCCGC

ataatgcttaagtcgaacagaaagtaatcgtattgtacacggccgcataatcgaaattaatacgactcactataggggaattgtgagcggataa
caattccccatcttagtatattagttaagtataagaaggagatatacatatgagcgataaaattattcacctgactgacgacagttttgacacggat
gtactcaaagcggacggggcgatcctcgtcgatttctgggcagagtggtgcggtccgtgcaaaatgatcgccccgattctggatgaaatcgct
gacgaatatcagggcaaactgaccgttgcaaaactgaacatcgatcaaaaccctggcactgcgccgaaatatggcatccgtggtatcccga
ctctgctgctgttcaaaaacggtgaagtggcggcaaccaaagtgggtgcactgtctaaaggtcagttgaaagagttcctcgacgctaacctgg
cgtaattaattaacctaggctgctgccaccgctgagcaataactagcataaccccttggggcctctaaacgggtcttgaggggttttttgctgaaa
ggaggaactatatccggattggcgaatgggacgcgccctgtagcggcgcattaagcgcggcgggtgtggtggttacgcgcagcgtgaccgc
tacacttgccagcgccctagcgcccgctcctttcgctttcttcccttcctttctcgccacgttcgccggctttccccgtcaagctctaaatcggggct
ccctttagggttccgatttagtgctttacggcacctcgaccccaaaaaacttgattagggtgatggttcacgtagtgggccatcgccctgatagac
ggtttttcgccctttgacgttggagtccacgttctttaatagtggactcttgttccaaactggaacaacactcaaccctatctcggtctattcttttgattta
taagggattttgccgatttcggcctattggttaaaaaatgagctgatttaacaaaaatttaacgcgaattttaacaaaatattaacgtttacaatttct
ggcggcacgatggcatgagattatcaaaaaggatcttcacctagatccttttaaattaaaaatgaagttttaaatcaatctaaagtatatatgagt
aaacttggtctgacagttaccaatgcttaatcagtgaggcacctatctcagcgatctgtctatttcgttcatccatagttgcctgactccccgtcgtgt
agataactacgatacgggagggcttaccatctggccccagtgctgcaatgataccgcgagacccacgctcaccggctccagatttatcagca
ataaaccagccagccggaagggccgagcgcagaagtggtcctgcaactttatccgcctccatccagtctattaattgttgccgggaagctaga
gtaagtagttcgccagttaatagtttgcgcaacgttgttgccattgctacaggcatcgtggtgtcacgctcgtcgtttggtatggcttcattcagctcc

ggttcccaacgatcaaggcgagttacatgatcccccatgttgtgcaaaaaagcggttagctccttcggtcctccgatcgttgtcagaagtaagttg
gccgcagtgttatcactcatggttatggcagcactgcataattctcttactgtcatgccatccgtaagatgcttttctgtgactggtgagtactcaacc
aagtcattctgagaatagtgtatgcggcgaccgagttgctcttgcccggcgtcaatacgggataataccgcgccacatagcagaactttaaaa
gtgctcatcattggaaaacgttcttcggggcgaaaactctcaaggatcttaccgctgttgagatccagttcgatgtaacccactcgtgcacccaa
ctgatcttcagcatcttttactttcaccagcgtttctgggtgagcaaaaacaggaaggcaaaatgccgcaaaaaagggaataagggcgacac
ggaaatgttgaatactcatactcttcctttttcaatcatgattgaagcatttatcagggttattgtctcatgagcggatacatatttgaatgtatttagaaa
aataaacaaataggtcatgaccaaaatcccttaacgtgagttttcgttccactgagcgtcagaccccgtagaaaagatcaaaggatcttcttga
gatcctttttttctgcgcgtaatctgctgcttgcaaacaaaaaaaccaccgctaccagcggtggtttgtttgccggatcaagagctaccaactcttttt
ccgaaggtaactggcttcagcagagcgcagataccaaatactgtccttctagtgtagccgtagttaggccaccacttcaagaactctgtagcac
cgcctacatacctcgctctgctaatcctgttaccagtggctgctgccagtggcgataagtcgtgtcttaccgggttggactcaagacgatagttac
cggataaggcgcagcggtcgggctgaacggggggttcgtgcacacagcccagcttggagcgaacgacctacaccgaactgagatacct a
cagcgtgagctatgagaaagcgccacgcttcccgaagggagaaaggcggacaggtatccggtaagcggcagggtcggaacaggagag
cgcacgagggagcttccagggggaaacgcctggtatctttatagtcctgtcgggtttcgccacctctgacttgagcgtcgatttttgtgatgctcgtc
aggggggcggagcctatggaaaaacgccagcaacgcggcctttttacggttcctggccttttgctggccttttgctcacatgttctttcctgcgttat
cccctgattctgtggataaccgtattaccgcctttgagtgagctgataccgctcgccgcagccgaacgaccgagcgcagcgagtcagtgagcg
aggaagcggaagagcgcctgatgcggtattttctccttacgcatctgtgcggtatttcacaccgcatatatggtgcactctcagtacaatctgctct
gatgccgcatagttaagccagtatacactccgctatcgctacgtgactgggtcatggctgcgccccgacacccgccaacacccgctgacgcg
ccctgacgggcttgtctgctcccggcatccgcttacagacaagctgtgaccgtctccgggagctgcatgtgtcagaggttttcaccgtcatcacc
gaaacgcgcgaggcagctgcggtaaagctcatcagcgtggtcgtgaagcgattcacagatgtctgcctgttcatccgcgtccagctcgttgagt
ttctccagaagcgttaatgtctggcttctgataaagcgggccatgttaagggcggttttttcctgtttggtcactgatgcctccgtgtaagggggatttc
tgttcatgggggtaatgataccgatgaaacgagagaggatgctcacgatacgggttactgatgatgaacatgcccggttactggaacgttgtga
gggtaaacaactggcggtatggatgcggcgggaccagagaaaaatcactcagggtcaatgccagcgcttcgttaatacagatgtaggtgttc
cacagggtagccagcagcatcctgcgatgcagatccggaacataatggtgcagggcgctgacttccgcgtttccagactttacgaaacacgg
aaaccgaagaccattcatgttgttgctcaggtcgcagacgttttgcagcagcagtcgcttcacgttcgctcgcgtatcggtgattcattctgctaac
cagtaaggcaaccccgccagcctagccgggtcctcaacgacaggagcacgatcatgctagtcatgccccgcgcccaccggaaggagctg
actgggttgaaggctctcaagggcatcggtcgagatcccggtgcctaatgagtgagctaacttacattaattgcgttgcgctcactgcccgctttc
cagtcgggaaacctgtcgtgccagctgcattaatgaatcggccaacgcgcggggagaggcggtttgcgtattgggcgccagggtggtttttcttt
tcaccagtgagacgggcaacagctgattgcccttcaccgcctggccctgagagagttgcagcaagcggtccacgctggtttgccccagcagg
cgaaaatcctgtttgatggtggttaacggcgggatataacatgagctgtcttcggtatcgtcgtatcccactaccgagatgtccgcaccaacgcg
cagcccggactcggtaatggcgcgcattgcgcccagcgccatctgatcgttggcaaccagcatcgcagtgggaacgatgccctcattcagca
tttgcatggtttgttgaaaaccggacatggcactccagtcgccttcccgttccgctatcggctgaatttgattgcgagtgagatatttatgccagcca
gccagacgcagacgcgccgagacagaacttaatgggcccgctaacagcgcgatttgctggtgacccaatgcgaccagatgctccacgccc
agtcgcgtaccgtcttcatgggagaaaataatactgttgatgggtgtctggtcagagacatcaagaaataacgccggaacattagtgcaggca
gcttccacagcaatggcatcctggtcatccagcggatagttaatgatcagcccactgacgcgttgcgcgagaagattgtgcaccgccgctttac
aggcttcgacgccgcttcgttctaccatcgacaccaccacgctggcacccagttgatcggcgcgagatttaatcgccgcgacaatttgcgacg
gcgcgtgcagggccagactggaggtggcaacgccaatcagcaacgactgtttgcccgccagttgttgtgccacgcggttgggaatgtaattca
gctccgccatcgccgcttccactttttcccgcgttttcgcagaaacgtggctggcctggttcaccacgcgggaaacggtctgataagagacacc
ggcatactctgcgacatcgtataacgttactggtttcacattcaccaccctgaattgactctcttccgggcgctatcatgccataccgcgaaaggttt
tgcgccattcgatggtgtccgggatctcgacgctctcccttatgcgactcctgcattaggaagcagcccagtagtaggttgaggccgttgagcac
cgccgccgcaaggaatggtgcatgcaaggagatggcgcccaacagtcccccggccacggggcctgccaccatacccacgccgaaacaa
gcgctcatgagcccgaagtggcgagcccgatcttccccatcggtgatgtcggcgatataggcgccagcaaccgcacctgtggcgccggtgat
gccggccacgatgcgtccggcgtagaggatcgagatcgatctcgatcccgcgaaattaatacgactcactata

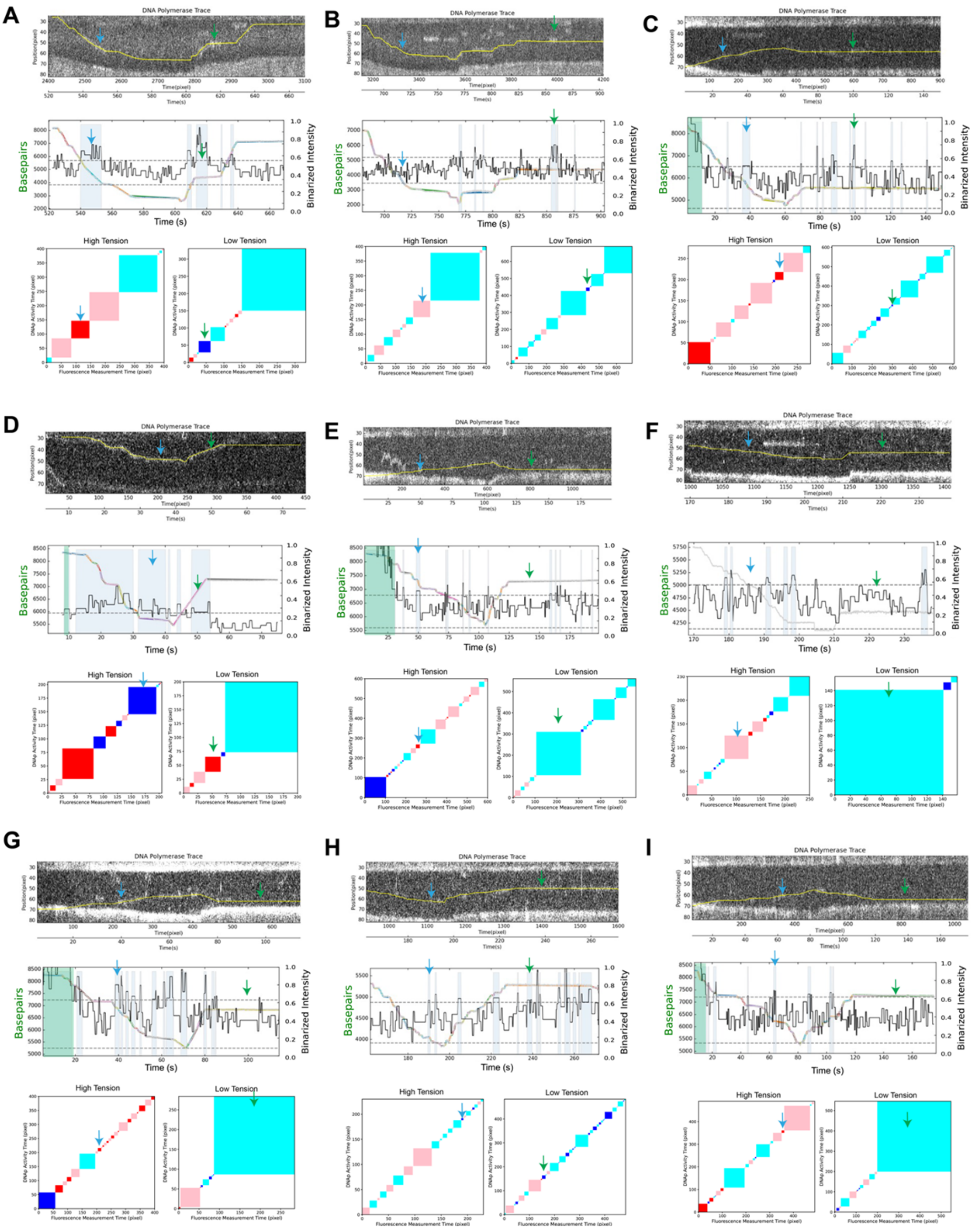

Supplementary Figure 1. Gallery of 9 distinct DNA polymerase traces (A-I). Each sub-figure's top panel displays overlapped DNA polymerase traces on the protein kymograph, the middle panel illustrates the correlated DNA polymerase traces and binarized fluorescence intensity, and the bottom panel shows the correlation heatmap of DNA polymerase activity and fluorescence intensity over time (see **Figure 1**). The blue and green arrows in each sub-figure highlights some of the fast-exchanging events in the DNA polymerase traces. Note, events near excessively bright trapped beads (example

as green shaded areas in C, E, G, I) were excluded from analysis, with 4 exo and 19 pol segments excluded for this reason. High-intensity bands at kymograph edges are from fluorescent beads used in the optical tweezer setup.

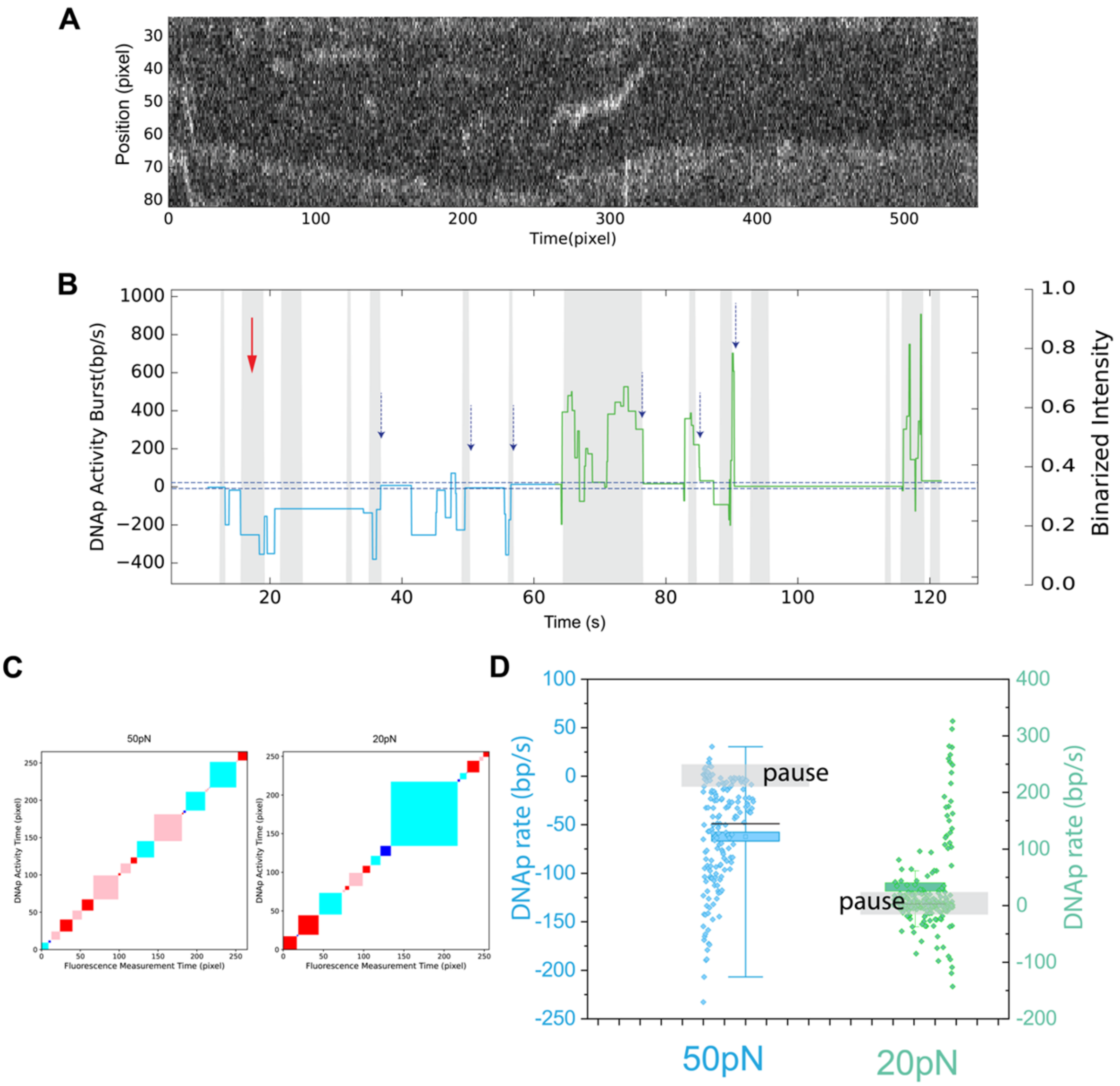

Supplementary Figure 2. Correlation Between Exonuclease and Polymerase Pausing Events with Fluorescence Intensity.

(**A**) Kymograph generated by recording fluorescence trajectories of DNA polymerases interacting with a DNA molecule over time. The data is derived from the same molecule as Figure 1E, but is presented here without the overlay to enhance clarity.

(**B**) Demonstrates the correlation between the stepwise burst activity of DNA polymerase and the binarized fluorescence signal. The respective exonuclease or polymerase rates of DNA polymerase are calculated using a step-fitting algorithm to pinpoint discrete burst activities, which are then related to the binarized fluorescence signal (see **Method**, **Figure 1G**). To ensure visual clarity, we incorporated two dashed horizontal lines (-10bp/s and 20bp/s), signifying the noise level standard deviation of the polymerase rates. The dashed horizontal lines demarcate the threshold between burst rate and pausing events - activities within this region are suggestive of pausing events. The

dashed purple arrows highlight instances where protein dissociation corresponds with a subsequent pausing event.

(**C**) Presents a correlation heatmap of DNA polymerase activity and fluorescence intensity under both 50pN and 20pN, using the same example as shown in **Figure 1H** for consistency.

(**D**) Depicts the burst rate of T7 DNA polymerase during 50 pN and 20 pN. The burst rate of DNA polymerase is determined as the average rate within a segment in single-molecule measurements, without distinguishing between single-type burst and transitional-type events (n = 177 exo segments and n = 202 pol segments). For the analysis of burst activities, events near excessively bright trapped beads (example in **Supplementary Figure 1**) were not excluded. Under the tension of 50 pN, DNA polymerase demonstrates an activity of -62 ± 4bp/s, whereas, at 20 pN, the activity is 32 ± 7bp/s, displaying a wide spread of rates. Note that these values are lower than those we have previously reported[8], as the mean rate within a segment does not exclude pausing events. The shadowed area indicates the occurrence of pausing events. Source data are provided as a Source Data file.

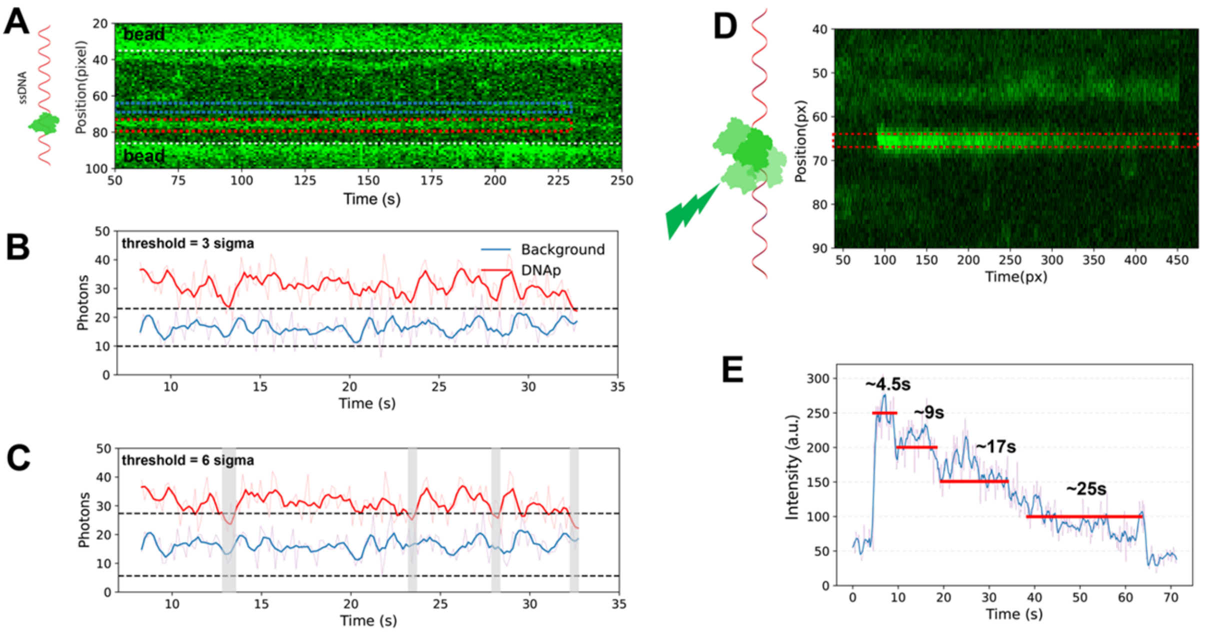

Supplementary Figure 3. Analysis of the photostability of DNA polymerase labelled with SNAP-Surface® 549.

(**A-C**) Evaluation of photoblinking of the fluorescently labelled DNA polymerase on a tensioned DNA substrate. (A) Representative kymograph trace of polymerase labelled with SNAP-Surface® 549 on on purely ssDNA. Dashed lines indicate bead edges, red rectangle shows 5-pixel polymerase trajectory region, purple rectangle is protein-free control region.The high-intensity bands at the top and bottom of the kymographs are due to fluorescence signals from the beads used in our optical tweezer's setup. (B) The DNA polymerase trace and background signal from (A) are extracted, with the raw data shown in light red and light purple, respectively, and filtered using an sg filter (shown in red and purple, respectively). The black dashed line indicates the 3-sigma threshold, which helps exclude background signals. In this case, the fluorescence signal fluctuates but remains above the background signal, ensuring it does not interfere with exchange analysis. (C) Shows the same data as in (B) but with a 6-sigma threshold, demonstrating over-filtering. When over-filtered, defined by the thresholding line exceeding the raw data, continuous protein data is split into segments. In our DNA polymerase exchange analysis, we use a 3-5 sigma threshold to avoid over-thresholding while efficiently filtering noise.

(**D-E**) Assessment of photobleaching of the fluorescently labelled DNA polymerase on a tensioned DNA substrate. (D) A representative trace of DNA polymerase labelled with SNAP-Surface® 549 on purely ssDNA, wherein labelled proteins are continuously scanned to bleach the fluorescent dye. (E) The corresponding fluorescence trace over time is extracted and depicted, demonstrating stepwise photobleaching of the protein. This is utilized to estimate the photobleaching duration. From the analysis of 17 bleaching steps from 5 distinct traces, an average photobleaching time of ~13s was determined under the specified imaging conditions. Notably, this calculated photobleaching time may underestimate the actual bleaching time due to the potential for protein dissociation. Source data are provided as a Source Data file.

A

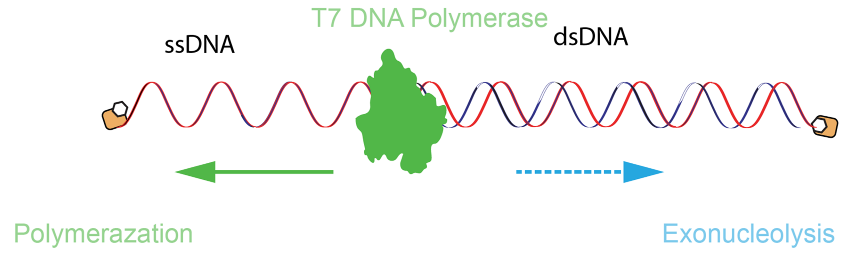

B

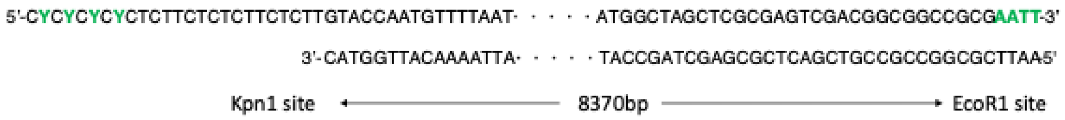

Supplementary Figure 4. Schematic of the DNA template (A) and sequence of PKYB 1 construct (B) used in the current study. The design of this DNA construct can be referred to publications[1]. The green letter in panel B indicated Biotinylated dATP used for bead tethering.

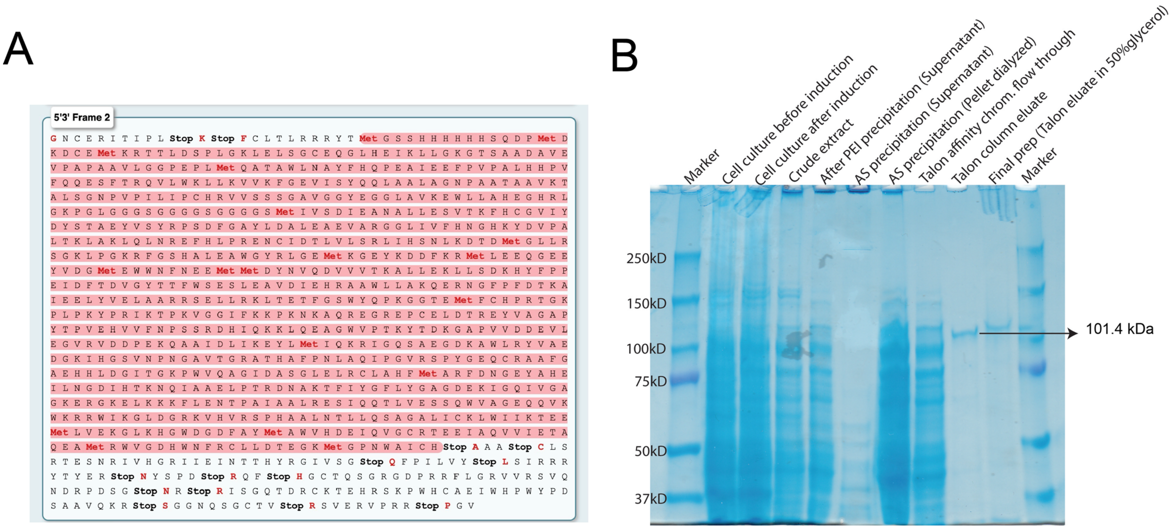

Supplementary Figure 5: Analysis of SNAP-DNA Polymerase Translation. (A) Protein translation prediction for the pETDuet-1_SNAP-DNA polymerase plasmid, using the Expasy Translation Tool from SIB Swiss Institute of Bioinformatics. (B) SDS-PAGE gel electrophoresis confirmed the molecular weight of SNAP-DNA polymerase (~100k Da).

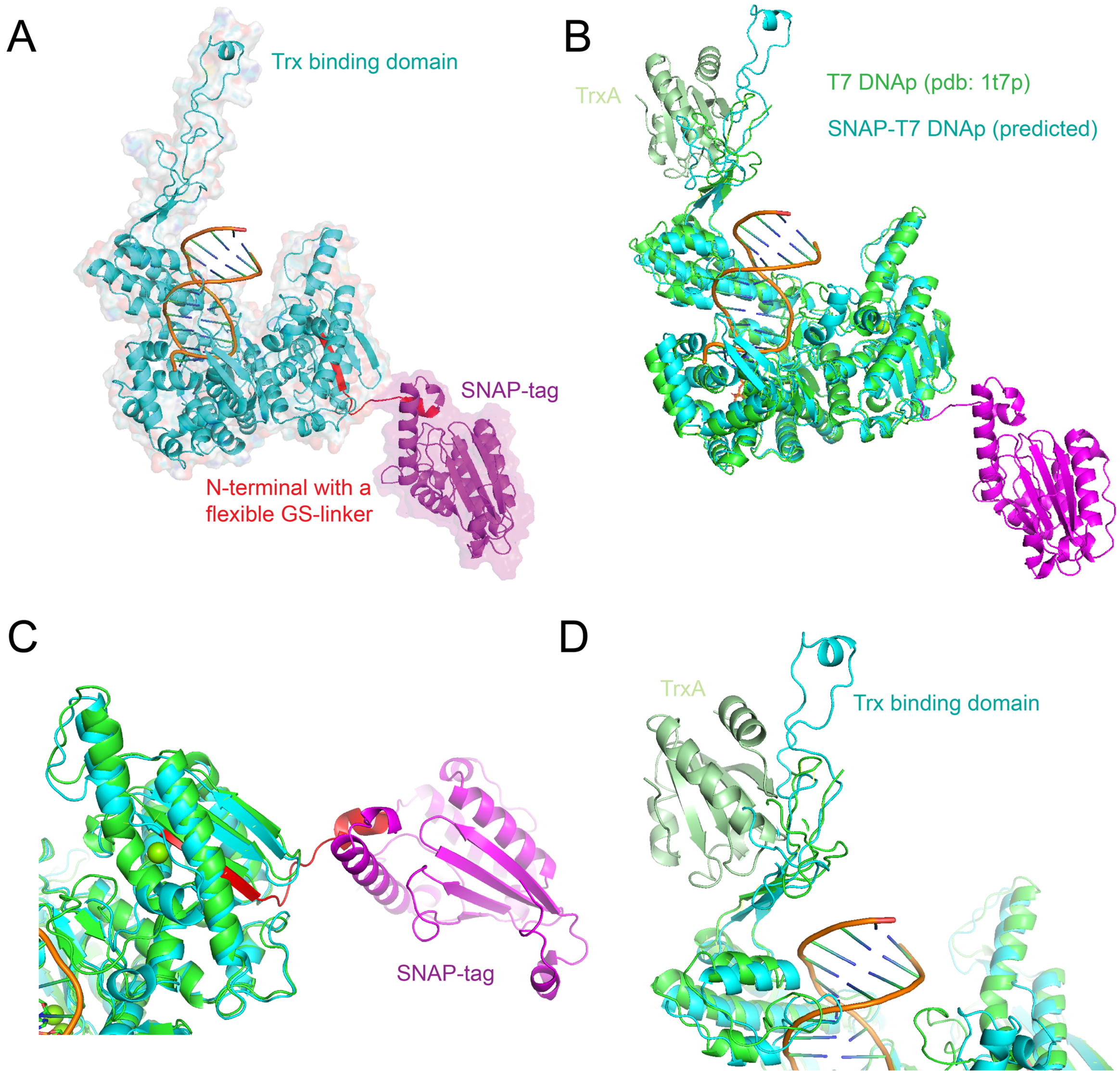

Supplementary Figure 6. Structure and Alignment of Predicted SNAP-DNA Polymerase with T7 DNA Polymerase. (A) Predicted structure of SNAP-DNA polymerase using the RoseTTAFold method[4], aligned with the experimentally determined T7 DNA polymerase (PDB:1T7P)[9] to model the complex with DNA primer/template. Color: SNAp-tag in magenta, N-terminal with flexible GS-linker in red, and gp5 protein in cyan. (B) Comparison of the RoseTTAFold-predicted SNAP-DNA polymerase structure with T7 DNA polymerase (PDB:1T7P), demonstrating preserved structure and active site for DNA binding. The experimental T7 polymerase is in green, with trxA indicated in light green. (C) Close-up of the N-terminal with a GS-linker and SNAP-tag, illustrating the tag's distance from the active site, suggesting minimal impact on binding and activity. (D) Zoomed-in view of the trxA-binding domain, showing its accessibility for trxA interaction.

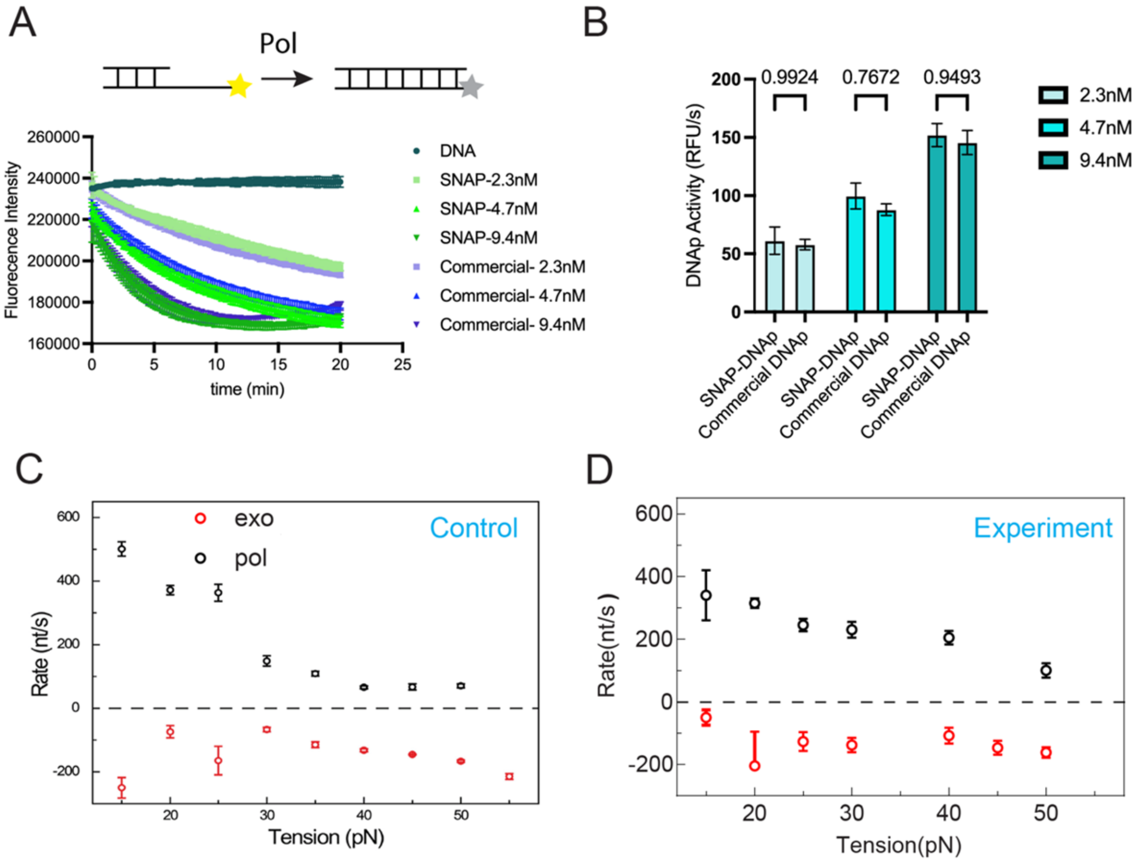

Supplementary Figure 7. **SNAP-DNAp exhibits activity comparable to commercial DNAp.** (**A**) Analysis of DNA polymerase activity using a real-time DNA primer extension assay reveals comparable performance between commercial T7 DNA polymerase (illustrated with a gradient of blue) and SNAP-DNAp pre-mixed with trx at a 1:4 ratio (shown with a gradient of green), across a range of enzyme concentrations (2.3nM, 4.7nM and 9.4nM). The assay measures the quenching of a 5′ fluorophore's intensity by nucleotide incorporation in the top strand. Data were plotted with three independent measurements. (**B**) Polymerase activity was quantified by analyzing the initial linear phase of the fluorescence intensity decrease (first 3 minutes). Values: mean ± sem, derived from three independent experiments. *p*-value is provided in the figure. Comparative Analysis of Polymerization and Exonucleolysis Activity Between Commercial T7 DNA Polymerase (Control, panel **C**) conducted in the same lab using same analysis method, adapted from ref [7] and Modified DNA Polymerase (Experiment, panels **D**).

References

1. Candelli, A. *et al.* A toolbox for generating single-stranded DNA in optical tweezers experiments. *Biopolymers* **99**, 611–620 (2013).
2. Johnson, D. E. & Richardson, C. C. A Covalent Linkage between the Gene 5 DNA Polymerase of Bacteriophage T7 andEscherichia coliThioredoxin, the Processivity Factor. *Journal of Biological Chemistry* **278**, 23762–23772 (2003).
3. Dangerfield, T. L., Kirmizialtin, S. & Johnson, K. A. Conformational dynamics during misincorporation and mismatch extension defined using a DNA polymerase with a fluorescent artificial amino acid. *Journal of Biological Chemistry* **298**, 101451 (2022).
4. Baek, M. *et al.* Accurate prediction of protein structures and interactions using a three-track neural network. *Science* **373**, 871–876 (2021).
5. Toste Rêgo, A., Holding, A. N., Kent, H. & Lamers, M. H. Architecture of the Pol III–clamp–exonuclease complex reveals key roles of the exonuclease subunit in processive DNA synthesis and repair. *EMBO J* **32**, 1334–1343 (2013).
6. Kerssemakers, J. W. J. *et al.* Assembly dynamics of microtubules at molecular resolution. *Nature* **442**, 709–712 (2006).
7. Hoekstra, T. P. *et al.* Switching between Exonucleolysis and Replication by T7 DNA Polymerase Ensures High Fidelity. *Biophysical Journal* **112**, 575–583 (2017).
8. Hoekstra, T. P. *et al.* Switching between Exonucleolysis and Replication by T7 DNA Polymerase Ensures High Fidelity. *Biophys J* **112**, 575–583 (2017).
9. Doublié, S., Tabor, S., Long, A. M., Richardson, C. C. & Ellenberger, T. Crystal structure of a bacteriophage T7 DNA replication complex at 2.2 Å resolution. *Nature* **391**, 251 (1998).
