# Supplementary material for: Mapping fast DNA polymerase exchange during replication
Source: Nat Commun. 2024 Jun 22;15:5328. doi: 10.1038/s41467-024-49612-3 (PMC11193749; doi:10.1038/s41467-024-49612-3)
Supplement: Supplementary file 3 — Reporting Summary [file 41467_2024_49612_MOESM3_ESM.pdf]

nature portfolio

Corresponding author(s): Gijs J.L. Wuite (g.j.l.wuite@vu.nl)

Last updated by author(s): Apr 25, 2024

Reporting Summary

Nature Portfolio wishes to improve the reproducibility of the work that we publish. This form provides structure for consistency and transparency in reporting. For further information on Nature Portfolio policies, see our Editorial Policies and the Editorial Policy Checklist.

Statistics

For all statistical analyses, confirm that the following items are present in the figure legend, table legend, main text, or Methods section.

n/a	Confirmed	
☐	☒	The exact sample size (*n*) for each experimental group/condition, given as a discrete number and unit of measurement
☐	☒	A statement on whether measurements were taken from distinct samples or whether the same sample was measured repeatedly
☒	☐	The statistical test(s) used AND whether they are one- or two-sided<br>*Only common tests should be described solely by name; describe more complex techniques in the Methods section.*
☒	☐	A description of all covariates tested
☒	☐	A description of any assumptions or corrections, such as tests of normality and adjustment for multiple comparisons
☐	☒	A full description of the statistical parameters including central tendency (e.g. means) or other basic estimates (e.g. regression coefficient) AND variation (e.g. standard deviation) or associated estimates of uncertainty (e.g. confidence intervals)
☒	☐	For null hypothesis testing, the test statistic (e.g. *F*, *t*, *r*) with confidence intervals, effect sizes, degrees of freedom and *P* value noted<br>*Give P values as exact values whenever suitable.*
☒	☐	For Bayesian analysis, information on the choice of priors and Markov chain Monte Carlo settings
☒	☐	For hierarchical and complex designs, identification of the appropriate level for tests and full reporting of outcomes
☒	☐	Estimates of effect sizes (e.g. Cohen's *d*, Pearson's *r*), indicating how they were calculated

Our web collection on statistics for biologists contains articles on many of the points above.

Software and code

Policy information about availability of computer code

Data collection	The data presented in the current study were collected using the LUMICKS C-Trap system in the .tdms format.
Data analysis	The data were analyzed using custom-written Python scripts, which are available for access at the following GitHub repository: https://github.com/longfuxu/DNAPolymeraseProject

For manuscripts utilizing custom algorithms or software that are central to the research but not yet described in published literature, software must be made available to editors and reviewers. We strongly encourage code deposition in a community repository (e.g. GitHub). See the Nature Portfolio guidelines for submitting code & software for further information.

Data

Policy information about availability of data

All manuscripts must include a data availability statement. This statement should provide the following information, where applicable:

- Accession codes, unique identifiers, or web links for publicly available datasets
- A description of any restrictions on data availability
- For clinical datasets or third party data, please ensure that the statement adheres to our policy

The datasets generated and/or analyzed during the current study has been submitted to Zenodo: https://doi.org/10.5281/zenodo.10782716

Research involving human participants, their data, or biological material

Policy information about studies with human participants or human data. See also policy information about sex, gender (identity/presentation), and sexual orientation and race, ethnicity and racism.

Reporting on sex and gender	Not Applicable - As this research did not involve human participants, there is no data on sex or gender to report.
Reporting on race, ethnicity, or other socially relevant groupings	Not Applicable - This study did not collect information on race, ethnicity, or any other socially relevant groupings.
Population characteristics	Not Applicable - No human populations were involved in the scope of this research.
Recruitment	Not Applicable - The study did not involve the recruitment of human participants.
Ethics oversight	Not Applicable - Given that there were no human participants, this study did not require ethical oversight in relation to human subjects.

Note that full information on the approval of the study protocol must also be provided in the manuscript.

Field-specific reporting

Please select the one below that is the best fit for your research. If you are not sure, read the appropriate sections before making your selection.

☒ Life sciences ☐ Behavioural & social sciences ☐ Ecological, evolutionary & environmental sciences

For a reference copy of the document with all sections, see nature.com/documents/nr-reporting-summary-flat.pdf

Life sciences study design

All studies must disclose on these points even when the disclosure is negative.

Sample size	For single-molecule experiments, data from 36 different DNA tethers confirm the observed phenotype, with 9 representative cases displayed in Figure S1. All data, currently available for review, will be publicly accessible upon conditionally accepted. The sample size was selected to achieve the statistical power needed to identify significant differences.
Data exclusions	In our single-molecule experiments, any tethers exhibiting atypical force-extension characteristics were excluded to ensure the integrity of our results. During fluorescence analysis, DNA constructs displaying protein aggregates were also omitted from the dataset. Protein events were excluded if they exhibited excessive brightness attributable to close proximity to the beads, as exemplified in Figure S1.
Replication	The presented data are consistently replicated across 36 independent DNA tethers, confirming the observed phenotype. DNA polymerase binding at ssDNA and dsDNA regiems are evidenced by 5-11 independent traces. Our findings, including all reported phenomena, are reproducible over several months, non-consecutive days, and across various protein batches.
Randomization	This does not apply for this study, since data in single-molecule studies are inherently randomized.
Blinding	In our single-molecule experimental set-up, blinding during data collection is inherently not possible. The integrity of our study is underpinned by objective, quantitative measures—specifically, the absolute end-to-end distance and counts of photons—rather than subjective assessments. Blinding was not implemented in the data analysis phase either. Nonetheless, standardized procedures for data collection and analysis were used to prevent bias.

Reporting for specific materials, systems and methods

We require information from authors about some types of materials, experimental systems and methods used in many studies. Here, indicate whether each material, system or method listed is relevant to your study. If you are not sure if a list item applies to your research, read the appropriate section before selecting a response.

Materials & experimental systems

n/a	Involved in the study
☒	☐ Antibodies
☒	☐ Eukaryotic cell lines
☒	☐ Palaeontology and archaeology
☒	☐ Animals and other organisms
☒	☐ Clinical data
☒	☐ Dual use research of concern
☒	☐ Plants

Methods

n/a	Involved in the study
☒	☐ ChIP-seq
☒	☐ Flow cytometry
☒	☐ MRI-based neuroimaging

Plants

Seed stocks

Not Applicable - This study did not involve the use of any seed stocks or other plant material.

Novel plant genotypes

Not Applicable - No novel plant genotypes were produced or analyzed as this study did not involve plant genetic materials or genotyping methods.

Authentication

Not Applicable - Since this study did not include plant materials, there were no authentication procedures conducted for seed stocks or novel genotypes.
